# Chronic and Acute Manipulation of Cortical Glutamate Transmission Induces Structural and Synaptic Changes in Co-cultured Striatal Neurons

**DOI:** 10.3389/fncel.2021.569031

**Published:** 2021-02-18

**Authors:** Naila Kuhlmann, Miriam Wagner Valladolid, Lucía Quesada-Ramírez, Matthew J. Farrer, Austen J. Milnerwood

**Affiliations:** ^1^Centre for Applied Neurogenetics (CAN), University of British Columbia, Vancouver, BC, Canada; ^2^Montreal Neurological Institute, Department of Neurology and Neurosurgery, McGill University, Montreal, QC, Canada; ^3^McKnight Brain Institute, University of Florida, Gainesville, FL, United States

**Keywords:** cortico-striatal co-culture, immunocytochemistry, electrophysiology, dendritic spines, synaptic plasticity, glutamate, long-term potentiation

## Abstract

In contrast to the prenatal topographic development of sensory cortices, striatal circuit organization is slow and requires the functional maturation of cortical and thalamic excitatory inputs throughout the first postnatal month. While mechanisms regulating synapse development and plasticity are quite well described at excitatory synapses of glutamatergic neurons in the neocortex, comparatively little is known of how this translates to glutamate synapses onto GABAergic neurons in the striatum. Here we investigate excitatory striatal synapse plasticity in an *in vitro* system, where glutamate can be studied in isolation from dopamine and other neuromodulators. We examined pre-and post-synaptic structural and functional plasticity in GABAergic striatal spiny projection neurons (SPNs), co-cultured with glutamatergic cortical neurons. After synapse formation, medium-term (24 h) TTX silencing increased the density of filopodia, and modestly decreased dendritic spine density, when assayed at 21 days *in vitro* (DIV). Spine reductions appeared to require residual spontaneous activation of ionotropic glutamate receptors. Conversely, chronic (14 days) TTX silencing markedly reduced spine density without any observed increase in filopodia density. Time-dependent, biphasic changes to the presynaptic marker Synapsin-1 were also observed, independent of residual spontaneous activity. Acute silencing (3 h) did not affect presynaptic markers or postsynaptic structures. To induce rapid, activity-dependent plasticity in striatal neurons, a chemical NMDA receptor-dependent “long-term potentiation (LTP)” paradigm was employed. Within 30 min, this increased spine and GluA1 cluster densities, and the percentage of spines containing GluA1 clusters, without altering the presynaptic signal. The results demonstrate that the growth and pruning of dendritic protrusions is an active process, requiring glutamate receptor activity in striatal projection neurons. Furthermore, NMDA receptor activation is sufficient to drive glutamatergic structural plasticity in SPNs, in the absence of dopamine or other neuromodulators.

## Introduction

The striatum is a highly integrative structure. In rodents, each of the ~2.5 million GABAergic medium-sized spiny projection neurons (SPNs) receives ~25,000 glutamate afferents from nearly all areas of the cortex and thalamus (Kincaid et al., 1998; Doig et al., 2010). These are modulated by nigrostriatal dopamine and form the only striatal output pathways (Tritsch and Sabatini, 2012). As the gateway to the basal ganglia, the striatum mediates action selection, motor control, motivation, and learning (Friend and Kravitz, 2014), and its dysfunction is implicated in neurodevelopmental disorders, addiction, and neurodegeneration (Graybiel et al., 2000; Smith et al., 2009; Gerfen and Surmeier, 2011). In contrast to the prenatal topographic development of sensory cortices, striatal circuit organization is slow and requires the functional maturation of cortical and thalamic excitatory inputs throughout the first postnatal month (Tepper et al., 1998). During this time, glutamate release promotes the formation and stabilization of excitatory synapses on SPNs (Kozorovitskiy et al., 2012, 2015). While mechanisms regulating synaptic development, maintenance, and plasticity are quite well described at glutamate synapses in principal excitatory neurons of the neocortex, much less is known of how these mechanisms translate to subcortical areas such as the striatum. Here, we developed assays to investigate excitatory synapse plasticity in a striatal *in vitro* system, where glutamate activity can be examined in isolation from dopamine and other neuromodulators.

Dendritic spines are specialized excitatory post-synaptic structures, which are thought to compartmentalize signaling processes to regulate glutamate receptor activation, calcium flux, cytoskeletal remodeling, membrane trafficking, and protein synthesis/degradation (Bourne and Harris, 2008; Yoshihara et al., 2009). Activity-dependent morphological changes in dendritic spines and associated presynaptic elements modulate neural function, with growth, pruning, and remodeling likely underlying cognitive processes (Villalba and Smith, 2013; Sala and Segal, 2014), and spine loss being a potential structural correlate of cognitive deficits (Penzes et al., 2011).

Excitatory synapse development, spine formation, and dynamics have been extensively studied in hippocampal/cortical pyramidal neurons, both *in vivo* and *in vitro*. Long-term potentiation (LTP) and long-term depression (LTD)-like paradigms have received particular attention, as these lasting activity-dependent modifications are considered the leading cellular model for learning and memory (Bliss and Collingridge, 1993). Spines alter shape and receptor composition in response to plasticity induction paradigms, with studies showing that LTP is typically associated with spine swelling (Matsuzaki et al., 2004; Okamoto et al., 2004; Tanaka et al., 2008) or *de novo* spine formation (Maletic-Savatic and Malinow, 1998; Engert and Bonhoeffer, 1999; Goldin et al., 2001), whereas LTD is associated with spine shrinkage (Okamoto et al., 2004; Zhou et al., 2004) or spine loss (Nägerl et al., 2004).

In recent genetic models where glutamate transmission is absent, principle excitatory neurons still develop normal dendritic architecture and spine numbers *in vivo* (Sando et al., 2017), a recent finding that adds weight to the traditional view that neuronal activity serves as a mechanism of refinement after synaptic connections are established (LeVay et al., 1980; Katz and Shatz, 1996; Sanes and Lichtman, 1999; Huberman et al., 2008). Conversely, there is evidence to support that activity is also important for synapse formation *per se* (Sabo et al., 2006; Andreae and Burrone, 2014; Choi B. J. et al., 2014; Choi S. H. et al., 2014; Okawa et al., 2014), reviewed in Andreae and Burrone (2018). In neuronal cultures, chronic action potential silencing throughout synaptogenesis reduces spine density by ~50% (Kossel et al., 1997), whereas after synapses form, a 3-day (but not 24 h) silencing period reduces spine number by only ~15% (Papa and Segal, 1996), suggesting that activity throughout the first 3 days contributes to synapse (and consequently spine) formation.

Alternatively, the observations that spine formation occurs on pyramidal neurons in the absence of vesicular glutamate release (Sando et al., 2017; Sigler et al., 2017), but is reduced in chronically-silenced cultures (Kossel et al., 1997), raise the possibility that early stages of spine formation may be regulated by GABAergic activity; inhibitory neurons are present in both scenarios, and GABA transmission (which is depolarizing in early development, and thus excitatory), is blocked in silenced cultures. Against this suggestion as a general rule, striatal cultures (almost entirely comprised of GABAergic SPNs) fail to generate appropriate dendritic arbors or dendritic spines in the absence of glutamatergic neurons, but do when co-cultured with cortical or hippocampal neurons (Segal et al., 2003; Kaufman et al., 2012; Fasano et al., 2013; Paraskevopoulou et al., 2019). Chronic silencing prevents spine formation even on co-cultured SPNs, which will develop spines within 2 h of TTX wash-out (Segal et al., 2003); this suggests connections are made despite silencing, and that spinogenesis specifically requires action potential-dependent transmission. Thus, SPNs require glutamatergic input to develop their eponymous morphology, and their dendritic spines appear to be highly plastic.

Alterations to striatal dendritic structures and synaptic plasticity are observed in multiple disorders, with evidence from many studies suggesting a role in the pathophysiology of Parkinson’s disease, Alzheimer’s disease, schizophrenia, and autism (McNeill et al., 1988; Day et al., 2006; Milnerwood and Raymond, 2010; Penzes et al., 2011; Villalba and Smith, 2013; Sala and Segal, 2014; Volta et al., 2017). Most knowledge of striatal plasticity comes from electrophysiological studies in acute brain slices; these have demonstrated a propensity to presynaptic plasticity and long-term depression, as well as the importance of neuromodulation by dopamine (Calabresi et al., 1997; Spencer and Murphy, 2000; Wang et al., 2006; Kreitzer and Malenka, 2007; Sergeeva et al., 2007; Li et al., 2009; Lovinger, 2010; Blackwell et al., 2019). That said, glutamate uncaging has also been shown to be sufficient to trigger *de novo* spine formation on SPN dendrites in ~50% of trials (Kozorovitskiy et al., 2012, 2015), similar to basal rates in cortical pyramidal neurons (Kwon and Sabatini, 2011). Elsewhere, Shen et al. (2008) demonstrated that dopamine was necessary for determining the directionality, but not necessarily the induction, of spike-time dependant plasticity in cultured striatal slices. Thus, glutamatergic modulation of striatal dendritic spines merits further attention, both during development and in response to activity-dependant plasticity.

Here, we examined how glutamate transmission, in the absence of dopamine, modulates SPN dendritic spine development, plasticity, and associated synaptic markers. Using an *in vitro* cortico-striatal co-culture system (Segal et al., 2003; Tian et al., 2010; Randall et al., 2011; Kaufman et al., 2012; Milnerwood et al., 2012; Penrod et al., 2015), we investigated the effects of blocking action potential-dependent network activity, manipulating spontaneous AMPA receptor (AMPAR) and NMDA receptor (NMDAR) activity, and the effects of an NMDAR-dependent LTP induction paradigm. Long-term glutamate silencing (>24 h) induced presynaptic alterations, reduced spine density, and had variable effects on filopodia, while short-term silencing (<3 h) did not. The LTP induction paradigm rapidly induced spine and GluA1 cluster changes, consistent with LTP-like modifications. We add to the literature by showing that glutamatergic activity is required for the maturation of striatal neurons, and demonstrate that glutamate receptor activity can induce structural plasticity in the absence of dopamine or other neuromodulators. The experiments here provide a foundation for future studies of activity-dependent striatal plasticity both in development and disease.

## Materials and Methods

### Culture Preparation

Wild-type (WT) C57BL/6J, and WT littermates from an LRRK2 G2019S knock-in colony (bred with the C57BL/6 colony, described in Beccano-Kelly et al., 2014) were maintained following the University of British Columbia animal care unit and the Canadian Council on Animal Care regulations. Primary neuronal cultures were prepared from mouse embryos (E16.5) of either sex. Briefly, brains were removed and dissected on ice in Hank’s Balanced Salt Solution (HBSS, GIBCO). For WT littermate cultures, tails were genotyped before cells were pooled, as in Beccano-Kelly et al. (2014). Cortical and striatal tissues were separately digested in 0.05% Trypsin-EDTA (LifeTech) at 37°C. Striatal cells were nucleofected with GFP on an AAV plasmid driven by a long-lasting (CAG/β-actin) promoter (pAAV-CAG-GFP; Addgene plasmid #37825): 1–2 million cells were suspended in 100 μl of electroporation buffer (Mirus Bio) with 1–2 μg of endonuclease-free DNA, transferred to a cuvette and electroporated using a Lonza Nucleofector 2b (Amaxa, program 05). The cell suspension was then removed and resuspended in plating medium (PM; 2% B27 + 1/100 penicillin/streptomycin, Invitrogen; 0.5 mM α-glutamine; neurobasal medium, GIBCO) and 24-well plates were seeded with non-transfected cortical neurons from the same mice at 1:1, to a density of 200,000 cells/well in 1 ml of PM. Cells were incubated at 37°C and 5% CO_2_, and, from days *in vitro* (DIV) 4 onwards, 10% of media was exchanged every 3–5 days until DIV21.

To verify survival and correct fluorophore/morphological identification of SPNs (as opposed to striatal interneurons and other cells) *in vitro*, additional co-cultures were prepared from homozygous BAC transgenic *Drd1a*-tomato mice [D1R-Tom, B6.Cg-Tg(Drd1a-tdTomato)6Calak/J, Jackson Laboratory, #016204], in which SPNs expressing the Drd1 dopamine receptor (D1R) are identified by Td-Tomato red fluorescent protein (Ade et al., 2011). To visualize SPNs expressing the Drd2 dopamine receptor (D2R), we used heterozygous *Drd2*-eGFP transgenic mice on an FVB/NJ background (D2R-eGFP, a gift from Raymond lab). Striatal or cortical neurons were nucleofected with TagBFP (pTagBFP-N; Axxora; EVN-FP172-C020) before plating 1:1 with non-nucleofected cells, and maintained until DIV21 as described above.

### Treatments

#### Chronic and Acute Action Potential Silencing

Co-cultures were left untreated 7 days after plating, to enable neurite outgrowth and synapse formation. Action potentials were then blocked by TTX application [1 μM; Tocris (IC_50_ ~7 nM)] in two ways: (A) throughout the rest of the 3-week development and maturation process, 3× TTX (TTX added at DIV7, 14 and 20); and (B) for the first 2 weeks with no further addition within the third week, 2× TTX (TTX at DIV7 and 14), and compared to control (sham; no drug added) neurons on the same 24-well plate. The concentration (far exceeding IC_50_) and time of TTX application was chosen to ensure effectiveness with fresh media addition (Takada et al., 2005; Hartman et al., 2006; Fishbein and Segal, 2011), and the 2× TTX (B) group included to see if a 7-day period is sufficient for recovery. One-hundred microliter of media was removed from each well and pooled by condition, then returned to each well, with, or without (sham) the addition of TTX.

Short-term disruption of glutamate signaling was achieved by blockade of burst firing with TTX (1 μM; Silencing), or of all excitatory activity (Total Silencing) by application of TTX, 6-cyano-7-nitroquinoxaline-2,3-dione disodium salt (CNQX; AMPA/kainite receptor antagonist; 10 μM; Tocris) and D-(−)-2-Amino-5-phosphonopentanoic acid (AP5; NMDA receptor antagonist; 10 μM; Tocris). At 24 or 3 h before fixation on DIV21, 100 μl of media was removed from each well and pooled by condition, then replaced with (for silencing) or without (sham control) drug addition.

#### Chemical Plasticity

The chemical long-term potentiation (cLTP) paradigm was achieved by applying glycine in the absence of extracellular magnesium (Mg^2+^), as previously described in hippocampal neurons (Lu et al., 2001; Brigidi et al., 2014). Briefly, media was removed from wells and replaced by an Mg^2+^-free extracellular solution (ECS; 125 mM NaCl, 33 mM D-glucose, 5 mM HEPES, 5 mM KCl, 2 mM CaCl_2_) containing 0.5 μM TTX and 20 μM bicuculline methiodide (10 mM stock; Tocris) for 15 min. One-hundred microliter of the solution was then removed from each well, 200 μM glycine (100 mM stock; Thermo Fisher Scientific) was added for cLTP condition and the solution was replaced, whereas removal/replacement without glycine addition acted as a negative sham control (cLTP Control). After 3 min, the solution in both groups was replaced with a fresh solution for 30 min, before fixation. A media removal and replacement group (without a change to Mg^2+^-free) acted as a second sham control.

### Immunostaining

Cells were fixed [4% Paraformaldehyde (PFA), 4% sucrose; 20 min], permeabilized [−20°C Methanol (MeOH) for 3 min] and blocked [3× 20 min wash with 10% normal goat serum (NGS) in phosphate-buffered saline (PBS), at room temperature (RT)]. Primary antibodies were incubated by shaking overnight at 4°C in PBS with Tween 20 (PBST) + 2% NGS, then cells were blocked again (10% NGS + PBS, 1 h RT) before secondary antibodies were applied (in PBST + 2% NGS, 30 min RT). Coverslips were washed (PBS, 3× 10 min) and slide-mounted with Fluoromount (Southern Biotech). The primary antibodies used were anti-GFP (Green Fluorescent Protein, mouse, Abcam Cat# ab1218 RRID: AB_298911, 1:1,000), anti-synapsin1 (Synapsin-1, rabbit, Millipore Cat# AB1543P RRID: AB_90757, 1:500), anti-GluA1 (AMPA Receptor, rabbit, Alomone Labs Cat# AGC-004 RRID: AB_2039878, 1:500), anti-tRFP (tagRFP, rabbit, Axxora Cat# EVN-AB233, 1:500). Secondary antibodies were anti-Mouse Alexa 488 (RRID: AB_2534069), anti-Rabbit Alexa 568 (RRID: AB_143157) and anti-Rabbit AMCA (all 1:1,000).

### Image Acquisition and Quantification

For co-culture characterization, 10–15 images were captured of each culture on an Olympus Fluoview 1000 confocal microscope (20× magnification, 1× confocal zoom), at random points across coverslips (for striatal marker co-expression counts) and targeted at BFP-expressing neurons (to verify D1R or D2R co-expression specifically with nucleofected neurons). The number of nucleofected neurons (blue) co-expressing Td-Tomato (red, D1R) or eGFP (green, D2R) were counted in ImageJ.

For other experiments, GFP-expressing neurons that fit D1R or D2R SPN morphology (Kaufman et al., 2012) were imaged as a series of 8–15 successive 0.5 μm *z*-stacks (60× oil immersion lens, 2× confocal zoom). Five to 10 SPNs were imaged per condition from a minimum of three independent cultures, with excitation and acquisition parameters constrained across all paired comparisons. The acquired images were sorted by channel and flattened using the max projection function on ImageJ for dendritic protrusion and cluster analysis.

For GluA1 and Synapsin-1 puncta analysis, images were manually thresholded and binarized by the eye using ImageJ, with the experimenter blind to condition. All quantification was conducted in Cell Profiler (http://www.cellprofiler.org; analysis pipeline included in Supplementary Material). Briefly, GFP-expressing cells were used to mask the dendritic arbor as the region of interest (ROI), which was then expanded by five pixels to capture apposing presynaptic elements. Binarized Synapsin-1 or GluA1 images were used to produce masks within the dendritic ROI, which was applied to the corresponding original (non-binarized) image to obtain puncta size (min diameter = 4 pixels; max = 15 pixels), intensity, and density (number of puncta/dendrite length) measures. Otsu’s method was used for automatic global thresholding of the images, and adjacent puncta were distinguished and divided by intensity.

To quantify dendritic protrusions, 3× ≥ 30 μm segments of secondary or tertiary dendrites, at least 30 μm from the soma were selected in the green channel (GFP fill) of each *z*-projected image in ImageJ. Dendrite length was recorded and manual 2D digital reconstruction was performed to count and measure each dendritic protrusion, with the experimenter blind to treatment condition. Protrusions were classified as either spine (<2 μm in length with a visible head >0.5 μm in diameter), or filopodia if they ranged between 1–10 μm and lacked a distinct bulbous head (Segal et al., 2003; Arstikaitis et al., 2011). The calculated densities and lengths for individual dendrites were averaged for a mean density per neuron. To quantify the percentage of spines associated with GluA1, clusters from binarized GluA1 images were manually counted within spines in three selected dendritic segments, excluding clusters that were clearly in perpendicular crossing neurites of other neurons.

Additional analysis was conducted on a large subset of images from chronic and acute silencing experiments, to quantify Synapsin-1 puncta on excitatory synapses only (those on spines and filopodia-like protrusions), as opposed to the entire dendrite masks. ImageJ was used to create ROIs around a sample of 20 spines (of varying shapes and widths) and any visible filopodia (ranging from 0 to 20) on secondary or tertiary dendrites of the GFP-expressing cell in each image. The ROIs were then applied as masks on the corresponding raw Synapsin-1 images, and the mean and integrated intensity measured within each ROI.

### Electrophysiology

Whole-cell voltage-clamp recordings were performed on GFP-expressing SPNs in the cortico-striatal co-cultures at DIV20–22 to measure functional changes following glycine application. 30 min after the cLTP or cLTP Control treatment, cells were perfused at room temperature with the extracellular solution (ECS) containing (in mM): 167 sodium chloride, 2.4 potassium chloride, one magnesium chloride, 10 glucose, 10 HEPES, two calcium chloride; pH 7.4, 290–300 mOsm. TTX (1 μM) and picrotoxin (PTX, 100 μM) were added to block spontaneous burst firing and GABAergic activity respectively. Pipette resistance (Rp) was 5–8 MΩ when filled with (in mM): 130 cesium methanesulfonate, five cesium chloride, four sodium chloride, one magnesium chloride, 5 EGTA, 10 HEPES, 5 QX-314, 0.5 GTP, 10 Na2-phosphocreatine, and 5 Mg ATP, 0.1 spermine; pH 7.3, 290 mOsm. The membrane test function was used to determine intrinsic membrane properties after obtaining whole-cell configuration, with a holding potential of −70 mV (Milnerwood et al., 2012). Following a 2-min settling period, miniature (spontaneously released, in the presence of TTX) excitatory post-synaptic currents (mEPSCs) were recorded at −70 mV. Data were acquired by Multiclamp 700 B amplifier and signals were filtered at 2 kHz, digitized at 10 kHz, and analyzed in Clampfit10 (Molecular Devices). Only recordings with a series resistance (Rs) <30 MΩ were included and ΔRs tolerance cut-off was <10%. mEPSCs were analyzed using the threshold search in Clampfit10 (threshold 5 pA) and additional visual quality control with the experimenter blind to genotype; monophasic events were used for amplitude and decay kinetics, while others were suppressed but included in frequency counts.

### Statistical Analysis

All statistical analyses were performed using Graphpad versions 7–9 (GraphPad software). For chronic and acute TTX experiments, spine/filopodia analysis is presented as raw data, whereas Synapsin-1 cluster data is normalized to the sham control within culture, to account for between-culture variation in immunostaining. Analyses were performed by one-way ANOVA and a Kruskal–Wallis test when data were not normally distributed (based on the d’Agostino and Pearson omnibus normality test). If significance was reached (at *p* < 0.05), *post-hoc* comparisons were made using uncorrected Fisher’s LSD (following one-way ANOVA) or uncorrected Dunn’s test (following Kruskal–Wallis). For chemical plasticity, the cLTP condition was normalized to cLTP control within culture, and comparisons made using two-tailed unpaired Student’s *t*-test, or the Mann–Whitney *U* test when data were not normally distributed. Statistical analyses are specified in each figure legend and all significant comparisons displayed by asterisks, with sample numbers (*n*) presented as number of images (number of independent cultures). Data are presented as mean ± SEM throughout.

## Results

### Characterization of Cortico-Striatal Co-cultures From D1R and D2R Reporter Mice

While striatal neurons develop poorly and have low viability in mono-culture (Segal et al., 2003; Kaufman et al., 2012; Burguière et al., 2013), those co-cultured with cortical neurons develop complex dendritic arbors and spines that stabilize around DIV20, and exhibit both morphological and electrophysiological properties resembling SPNs *in vivo* (Segal et al., 2003; Tian et al., 2010; Randall et al., 2011; Kaufman et al., 2012; Milnerwood et al., 2012; Burguière et al., 2013; Lalchandani et al., 2013; Penrod et al., 2015). Over 95% of total striatal cells are SPNs *in vivo* (Kawaguchi and Kubota, 1997), a proportion that is maintained *in vitro* (Shehadeh et al., 2006). Of these, ~half express the D1 dopamine receptor, and half express the D2 dopamine receptor (Kreitzer, 2009).

**Figure 1:**
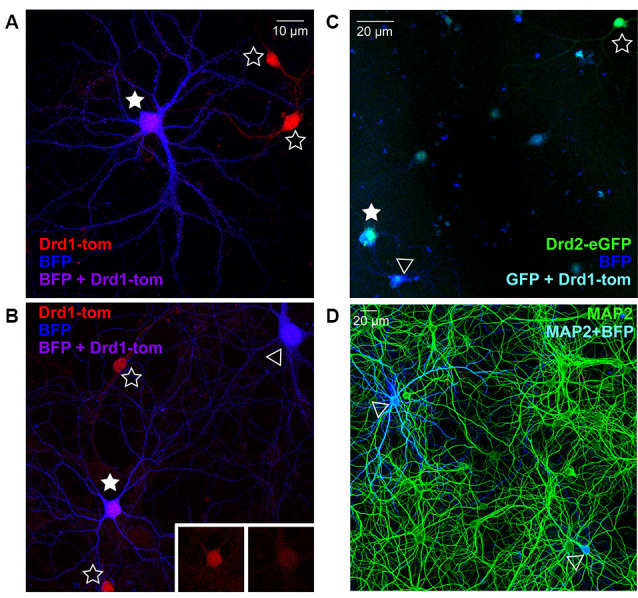
Nucleofection and spiny projection neuron (SPN) identification in co-cultures from reporter line mice. **(A,B)** Representative images of days *in vitro* (DIV) 21 striatal neurons from *Drd1a*-tdTomato (D1R) BAC transgenic reporter line mice, nucleofected with BFP expression constructs and grown in co-culture with cortical neurons (20× magnification, 2× zoom Olympus FV-1000). **(A)** D1R SPN (red) co-labeled with BFP-fill (blue) is shown (purple; filled star), surrounded by two non-nucleofected (open star) D1R SPNs. **(B)** Two BFP filled SPNs are apparent, one of which is D1R+ve (filled star) and one is negative (open arrowhead), as apparent in somatic D1 signal (inserts); two D1R+ve non-nucleofected SPNs are nearby (open stars). **(C)** Representative image of DIV21 striatal neurons from *Drd2*-eGFP (D2R) BAC transgenic reporter line mice, nucleofected with BFP expression constructs and grown in co-culture with cortical neurons (20× magnification, Olympus FV-1000). A D2R SPN (green) co-labeled with BFP-fill (blue) is shown (cyan; filled star), near to a non-nucleofected (open star) D2R SPN. A BFP filled SPN is apparent, which is D2R+ve (open arrowhead). **(D)** Representative image (20× magnification) of DIV21 cortico-striatal co-cultures from non-transgenic mice. Striatal neurons nucelofected with BFP expression constructs (blue) before plating, and cultures were stained for MAP2 (green) to verify the density of nucleofected striatal neurons. Two BFP-expressing neurons are visible (open arrowheads).

To verify the nucleofection of isolated striatal cells before mixing in co-culture, and ensure correct visual identification of SPNs by the experimenter based on fluorescent fills, we quantified the co-expression of BFP plasmid-nucleofected striatal neurons in cultures prepared from germ-line SPN marker mice; for D1R SPNs we used *Drd1a-*tdTomato reporter mice, and for D2R SPNs we used Drd2-eGFP reporter mice. BFP-expressing neurons showing characteristic SPN morphology, as described in previous studies (Kaufman et al., 2012; Burguière et al., 2013), were imaged before checking for D1R or D2R co-expression, to test the accuracy of the experimental assessment. Given that >95% of striatal cells are SPNs, D1- and D2-expressing cells should each account for ~50% of all BFP-expressing neurons; however, it should be noted that in acute slices and cultures, the segregation in double-fluorophore mice is 60% D1 vs. 40% D2 (Thibault et al., 2013). In line with this, 56% of imaged BFP neurons (in two separate cultures) in cultures from Drd1a-tdTomato reporter mice were Drd1 positive (Figures 1A,B). In cultures from Drd2-eGFP reporter mice (Figure 1C), ~28% of BFP-expressing neurons (over two separate cultures) co-expressed D2R, slightly below the expected proportion. Together the results demonstrate that at least 80% of BFP-filled cells are clearly identified as SPNs, based on BAC fluorophore expression.

### Chronic TTX Application During Development Alters Dendritic Protrusions and Synapsin-1 Clusters in SPNs

**Figure 2:**
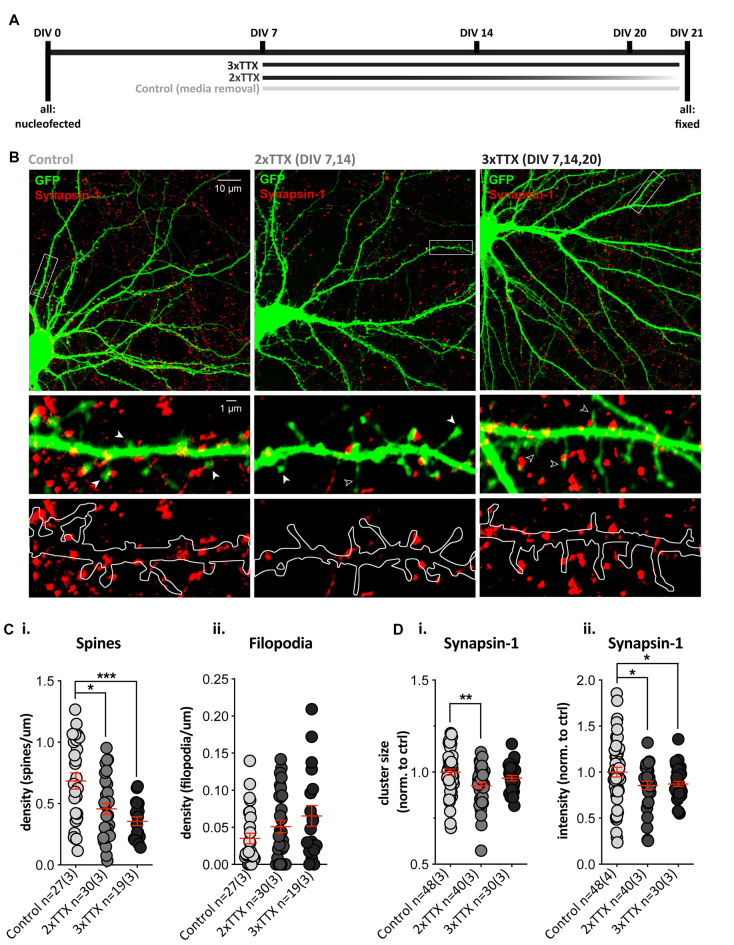
Chronic TTX treatment decreases spine density and Synapsin-1 intensity. GFP-expressing striatal neurons were grown in co-culture at a 1:1 ratio with cortical cells until DIV21, then fixed and stained with anti-GFP (green) and the presynaptic terminal marker Synapsin-1 (red). **(A)** Experimental timeline for each condition. Cells were treated with 3× TTX (DIV 7, 14 and 20), 2× TTX (DIV 7&14), or received a media removal sham treatment (Control). **(B)** Top: representative images for each condition (Olympus FV-1000, 60×, 2× zoom). Middle and bottom: expanded images of the dendritic segment marked by the white rectangle in the corresponding top image (digital zoom). Overlay of GFP fill and Synapsin-1 staining (middle) showing dendritic spines (filled arrowhead), filopodia (open arrowhead), and Synapsin-1-positive presynaptic terminals in red. Outline of GFP filled with Synapsin-1-positive puncta (bottom) to show presynaptic terminals and masked area for quantification. **(Ci)** There was a significant decrease in dendritic spine density (averaged across three quantified dendritic segments per neuron) in both chronic TTX treatments (Kruskal–Wallis test, **p = 0.002; *post-hoc* 2× TTX, *p = 0.019, 3× TTX, ***p = 0.0007) relative to control, but there was no significant difference between the two TTX treatments (*p* = 0.188). **(ii)** Filopodia density was not significantly increased following TTX treatment relative to control (Kruskal–Wallis test, *p* = 0.155). **(Di,ii)** Synapsin-1 cluster size was only significantly reduced in the 2× TTX condition compared to control (i; Kruskal–Wallis test, **p = 0.008, *post-hoc* 2× TTX, **p = 0.002, 3× TTX, *p* = 0.147), and there was no significant difference between treatment groups (*p* = 0.179). Synapsin-1 cluster intensity was reduced in both TTX conditions relative to the control (ii; one-way ANOVA, *F*_(2,115)_ = 3.409, *p = 0.036; *post-hoc*, 2× TTX, *p = 0.026 and 3× TTX, *p = 0.034), and there was no difference between the two TTX treatment groups (*p* = 0.791).

The role of bursts of synchronous (action potential-mediated) excitatory release onto SPNs during synapse maturation was assessed by chronic blockade of action potentials (TTX) in cortico-striatal co-cultures over 2 (2× TTX, at DIV 7, 14) or 3 (3× TTX, at DIV 7, 14 and 20) weeks (Figure 2A). Quantification of dendritic protrusions on GFP-expressing SPNs (Figure 2B) revealed a significant effect of treatment upon SPN spines (Figure 2Ci), with *post-hoc* analysis demonstrating significantly lower density in both TTX-treated groups, relative to untreated SPNs (control = 0.68 ± 0.07, 2× TTX = 0.46 ± 0.05, and 3× TTX = 0.36 ± 0.04 spines/μm dendrite). While filopodia density was not significantly altered, there was a clear trend toward TTX treatment increasing filopodia in a dose/time-dependent manner (Figure 2Cii).

To assess whether postsynaptic structural alterations were associated with a change in presynaptic contacts, we quantified Synapsin-1 clusters (present at both glutamatergic and GABAergic synapses) in contact with dendrites on GFP-filled SPNs. The density of Synapsin-1 clusters did not differ between treatment groups (Supplementary Figure 1A), but there was a significant main effect of treatment upon cluster size (Figure 2Di) and cluster intensity (Figure 2Dii), with both TTX-treated groups showing a reduction when normalized to control SPN values (2× TTX = 0.85 ± 0.05 and 3× TTX = 0.87 ± 0.16). The size of Synapsin-1 clusters was only significantly reduced compared to control SPNs in the 2× TTX condition (Figure 2Di; 0.93 ± 0.02). To verify that the observed changes occurred at excitatory synapses, we quantified puncta signal intensity directly on spines and filopodia (Supplementary Figure 1A); in agreement with reduced presynaptic intensity on whole dendritic masks, Synapsin-1 signal was significantly reduced on spines in cultures that were silenced for the full period (3× TTX), relative to both untreated and transiently-silenced cultures (2× TTX, *p* < 0.0001 and *p* = 0.008, respectively). While not statistically significant, there was an intermediate reduction in Synapsin-1 signal on spines in the transiently-silenced group. This demonstrates that presynaptic alterations in Synapsin-1 signal on spines (Supplementary Figure 1A) correlate with the reductions to spine density (Figure 2Ci) and Synapsin-1 signal on whole dendrite masks (Figure 2D). Silencing duration gradually increased the density of filopodia, but not significantly (Figure 2Cii), and while Synapsin-1 signal was unaltered on filopodia of cultured SPNs silenced for the full period, in the transiently-silenced group Synapsin-1 signal was significantly increased, relative to both untreated and total silenced (Supplementary Figure 1A). This suggests that the overall reduction in presynaptic signals onto SPN dendrites of silenced cultures is predominantly at more mature dendritic spines and that transient silencing results in an increase in presynaptic signal on filopodia (and a rebound increase in spines) following removal of TTX.

Together the data demonstrate that sustained chronic blockade of burst firing in cortico-striatal co-cultures alters presynaptic inputs, in concert reducing the density of dendritic spines by either: (1) preventing spine formation in SPNs (which recovers partly when TTX is removed); or (2) causing a gradual loss of spines that is more pronounced with longer silencing.

### Glutamatergic Silencing Alters Dendritic Protrusions and Synapsin-1 Clusters After 24, but Not 3, Hours

**Figure 3:**
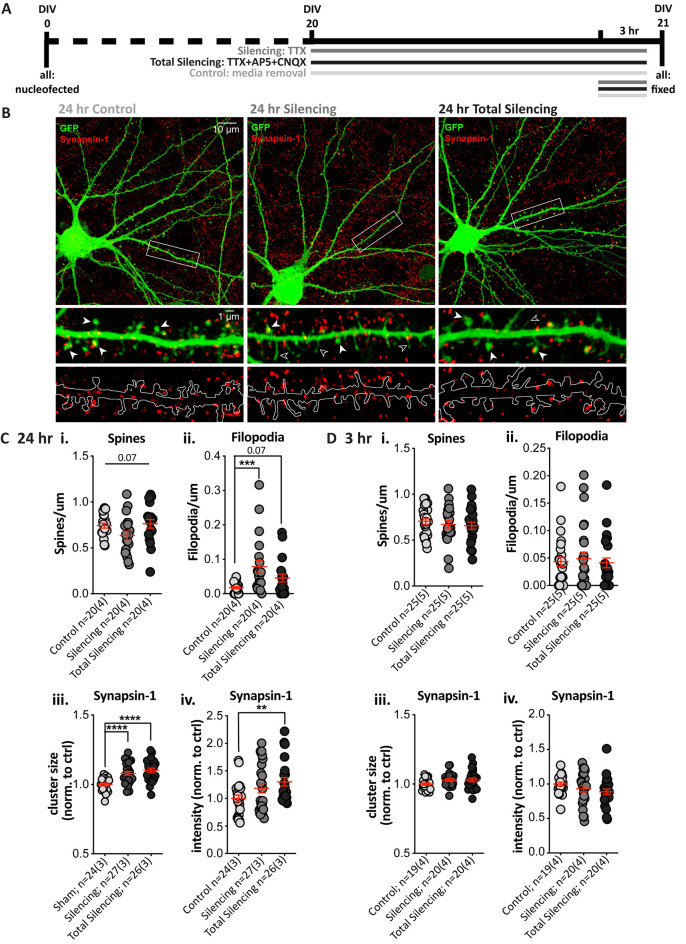
Blocking glutamatergic activity induces structural changes and increases Synapsin-1 cluster intensity after 24, but not 3, hours. **(A)** Experimental timeline for each condition. TTX (Silencing) or TTX + AP5 + CNQX (Total Silencing) was administered either 24 or 3 h before fixation on 21 days *in vitro* (DIV). **(B)** Top: representative cell images from each condition (Olympus FV-1000, 60×, 2× zoom). Middle and bottom: expanded images of the dendritic segment outlined in the white rectangle (digital zoom), showing the GFP fill (green) and Synapsin-1-positive presynaptic terminals (red, middle) with visible spines (filled arrowhead) and filopodia (open arrowhead), and an outline of the GFP fill with Synapsin-1 puncta (bottom) to illustrate the masked area for quantification. **(C)** Results from 24 h blockade. **(i)** There was no significant difference in spine density, despite a strong trend to a reduction following TTX treatment (one-way ANOVA, *F*_(2,57)_ = 2.812, **p = 0.068). **(ii)** There was a significant increase in filopodia density following TTX treatment as compared to control cells (Kruskal–Wallis test, **p = 0.004, *post-hoc* ***p = 0.0008), which was not observed when AMPA and NMDA receptors were also blocked (*p* = 0.068) and *post-hoc* comparisons revealed no significant difference between the TTX treatment groups (*p* = 0.127). **(iii,iv)** Synapsin-1 cluster size was significantly increased in both silencing conditions relative to control. **(iii)** One-way ANOVA, *F*_(2,74)_ = 15.617, ****p < 0.000001; *post-hoc* Silencing ****p < 0.0001 and Total Silencing ****p < 0.000001); and there was no significant difference between treatment groups (*p* = 0.227). The integrated intensity was only significantly increased in the Total Silencing condition compared to control (iv; Kruskal–Wallis test, *p = 0.024, the *post-hoc* *p* value is referred to on the graph **p = 0.007), whereas the Silencing condition did not differ significantly either from control (*p* = 0.230) or Total Silencing conditions (*p* = 0.115). **(Di–iv)** No significant changes in dendritic protrusions or Synapsin-1 clusters were observed following a 3 h treatment. **(i)** Spines *p =* 0.6. **(ii)** Filopodia *p* = 0.9. **(iii)** Size *p* = 0.1. **(iv)** Intensity *p* = 0.1.

Next, we tested whether pre-and post-synaptic changes would still be observed following a shorter (24 h) TTX application (Silencing), and after additionally using antagonists to block glutamate signaling from action potential-independent (miniature/spontaneous) release and AMPA and NMDA receptor signaling (Total Silencing, Figure 3A). A treatment effect on spine density in GFP-filled SPNs (Figure 3B) neared statistical significance (Figure 3Ci, *p* = 0.07), due to reduced spine density in the TTX Silencing group, whereas Total silencing appeared to prevent this. There was a significant main effect of treatment upon filopodia density, with a significant increase following TTX application only, and a strong trend to an increase following total silencing (Figure 3Cii).

Analysis of Synapsin-1 revealed that cluster density was not altered (Supplementary Figure 1B), but cluster size was significantly increased in both the Silencing and Total Silencing conditions when normalized to control SPNs (Figure 3Ciii, 1.08 ± 0.01, *p* < 0.0001 and 1.10 ± 0.02, respectively). Cluster intensity was also significantly increased after Total Silencing, whereas there was only a trend to increase in the Silencing condition (Figure 3Civ). Analysis of Synapsin-1 signal specifically on dendritic spines and filopodia demonstrated an increase in spines similar to that observed on whole dendrite masks, but no change on filopodia (Supplementary Figure 1B). The data suggest presynaptic alterations (increased Synapsin-1 signal) occur on all spines upon TTX silencing, before robust spine elimination by longer (chronic) TTX silencing; this spine loss is prevented by blocking residual spontaneous activity in the Total Silencing group, despite similar presynaptic effects. Conversely, no presynaptic changes were detected on filopodia, despite their increased density, which must require more sustained (chronic) silencing.

A shorter 3 h blockade of glutamatergic activity produced no significant differences in spine density (Figure 3Di), filopodia density (Figure 3Dii), or any measures of Synapsin-1 clusters (Figures 3Diii,iv). Thus, a 3 h silencing period is insufficient to drive structural changes in SPNs, whereas a 24 h silencing period causes pre- and post-synaptic changes. Interestingly, blocking AMPA and NMDA receptors prevented dendritic protrusion changes, but not Synapsin-1 signal increases. The results suggest that postsynaptic structural plasticity is dependent upon residual, presumably miniature, NMDA/AMPA receptor glutamate signaling over a 24 h period; in contrast, presynaptic alterations are apparent in response to silencing at terminals on spines (but not filopodia), regardless of NMDA and AMPA receptor signaling.

### Chemical LTP Significantly Increases Spine Density, GluA1 Expression, and Alters mEPSC Properties in SPNs

**Figure 4:**
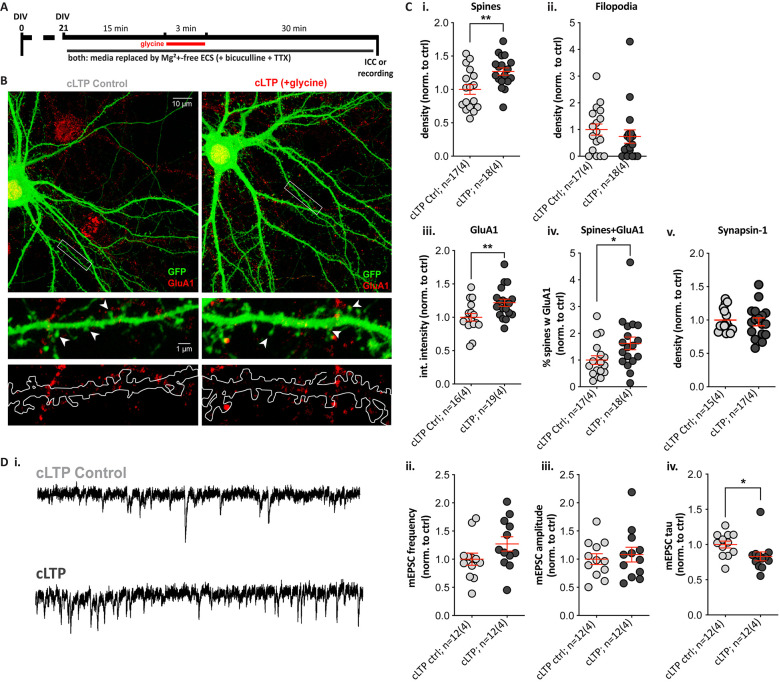
A 3-min glycine cLTP induction protocol induced spine density and GluA1 cluster increases within 30 min and altered the decay time of miniature events. **(A)** Experimental timeline. Following glycine or control treatment, cells were fixed and immunostained with anti-GFP (green) and the presynaptic terminal marker Synapsin-1 (not shown) or the postsynaptic AMPA receptor subunit GluA1 (red). Separate coverslips were used for whole-cell voltage-clamp recordings. **(B)** Top: representative images (Olympus FV-1000, 60×, 2× zoom) of control (cLTP ctrl) and +glycine (cLTP) cells. Middle and bottom: expanded images (digital zoom) depicting the dendritic segment outlined in the white rectangle above. Middle row images show GFP fill with visible spines and GluA1 clusters, and in the lower panel, the GFP fill is outlined to depict the masked area for puncta quantification. **(C)** Structural and synaptic marker changes following glycine treatment in Mg^2+^-free ECS. **(i,ii)** The analysis revealed a ~30% increase in spine density relative to control-treated SPNs. **(i)** Unpaired *t*-test, **p = 0.006; whereas no change was observed in filopodia density. **(ii)** *p* = 0.2. **(iii,iv)** GluA1 cluster intensity was significantly increased in glycine-treated relative to control SPNs. **(iii)** Unpaired *t*-test, **p = 0.009; as was the percentage of spines colocalized with GluA1 clusters in glycine treated SPNs. **(iv)** Mann–Whitney test, *p = 0.022. **(v)** No changes in Synapsin-1 cluster density were observed. **(D)** Whole-cell patch-clamp recordings from cLTP and control SPNs. **(i)** Representative traces showing miniature excitatory postsynaptic currents (mEPSCs) in the control (top) and glycine-treated (bottom) SPN. **(ii,iii)** Despite a trend, there was no significant difference in mEPSC frequency. **(ii)** *p* = 0.1 and no change in amplitudes. **(iii)** *p =* 0.5. **(iv)** The mEPSC decay time (tau) was significantly faster, following glycine treatment (Mann–Whitney test, *p = 0.015).

To determine whether striatal SPNs can exhibit LTP-like changes without the contribution of neuromodulators, we used a pharmacological induction paradigm for NMDAR-dependent LTP with the NMDAR co-agonist glycine, a protocol similar to what we and others have previously used in cultured hippocampal neurons (Park et al., 2006; Fortin et al., 2010; Brigidi et al., 2014). Cells were treated with glycine for 3 min in Mg^2+^-free extracellular solution (cLTP), using a switch to Mg^2+^-free solution with no glycine addition as a control condition (cLTP control; Figure 4A). There were no significant differences between the cLTP control and the sham control (media removal and replacement; data not shown), and cLTP results were normalized to cLTP control within each culture. Quantification of dendritic protrusions on GFP-expressing SPNs (Figure 4B) revealed that glycine treatment resulted in a significant ~30% increase in spine density (Figure 4Ci; cLTP ctrl = 1.00 ± 0.07 and cLTP = 1.27 ± 0.06), with no change in filopodia density (Figure 4Cii). Additionally, cLTP treatment resulted in a significant increase in GluA1 cluster intensity compared to control SPNs (Figure 4Ciii, 1.00 ± 0.06 and 1.22 ± 0.05 respectively) but no difference in GluA1 cluster density or size (data not shown). Quantification of the percentage of dendritic spines containing clear GluA1 clusters was significantly higher (70% increased) in glycine-treated cultures (Figure 4Civ). Presynaptic Synapsin-1 staining did not change following glycine treatment (Figure 4Cv).

Since glycine treatment increased spine density and GluA1 signal, we next assessed functional effects by whole-cell voltage-clamp recordings of miniature excitatory postsynaptic currents (mEPSCs) in control and glycine-treated GFP-filled SPNs (Figure 4Di). Increases in mEPSC frequency (reflective of increased presynaptic glutamate release or active synapses) and increased amplitude (postsynaptic responsiveness) are detected in glycine-treated hippocampal neurons (Brigidi et al., 2014). The frequency of mEPSCs appeared ~20% higher in glycine-treated SPNs, but the trend was not significant (Figure 4Dii), and there were no trends to increased mEPSC amplitude (Figure 4Diii). However, mEPSC event decay time constants (tau) were significantly faster in cLTP SPNs (Figure 4Div); this was not explained by passive membrane properties, which did not differ between the two groups (data not shown). These results suggest that NMDAR activation alone is sufficient to drive LTP-like structural changes in SPNs and to induce significant alterations to the properties of excitatory currents.

## Discussion

**Figure 5:**
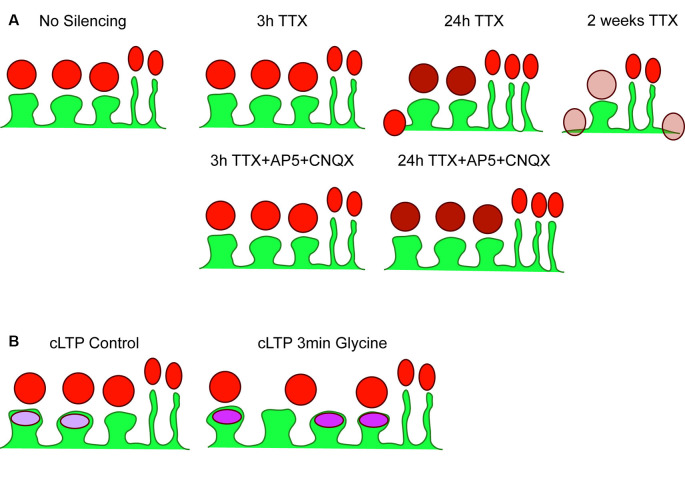
Summary of results. **(A)** Cartoon depicting basal (no silencing) striatal projection neuron dendrite (SPN; green) with three postsynaptic spines and two filopodia, with presynaptic Synapsin-1 signal shown in red. There were no changes detected following 3 h TTX silencing or 3 h TTX + AP5+CNQX. Silencing with TTX for 24 h increased Synapsin-1 signal intensity specifically on dendritic spines (darker red), and began the process of spine elimination, without changes to filopodia density. The additional block of spontaneous glutamate receptor activation (AP5 + CNQX) prevented reductions in spine density but not increased Synapsin-1 signal intensity. Chronic silencing with 2-week exposure to TTX dramatically reduced spine density and decreased Synapsin-1 signal intensity; specifically, that associated with dendritic spines and dendrites. **(B)** Cartoon of control (no glycine) SPN with three spines and two filopodia with postsynaptic GluA1 AMPA receptor signal shown in purple. A chemical LTP protocol (3 min glycine) increased spine density, GluA1 signal intensity, and the percentage of spines with clearly detected GluA1 clusters, without changing presynaptic cluster density.

The structural responses of SPNs to altered glutamate input were examined within the context of cortico-striatal co-cultures, and findings are summarized in the graphical abstract (Figure 5). A reduction in spine density following chronic or medium-term TTX application suggests that excitatory action potential firing in cortico-striatal networks is a crucial regulator of dendritic spines, and thus of excitatory synapse development and/or maintenance on striatal neurons; however, a contributing role of GABA from interneurons or SPNs themselves cannot be ruled out in the present work. NMDAR-dependent structural LTP-like changes were rapidly induced by glycine stimulation, as evidenced by a ~30% increase in spine density and GluA1 cluster signals within 30 min. Overall, these pharmacological silencing and plasticity experiments indicate that altering glutamatergic activity is sufficient to drive structural plasticity in SPNs, even in the absence of dopamine and other striatal neuromodulators. Furthermore, while there is an ongoing debate about the role of filopodia as intermediates in spine formation (reviewed in Sala and Segal, 2014), our finding that spine and filopodia densities were not always negatively correlated supports the notion that they are, at least in part, regulated by distinct processes.

We replicate a previous finding that the continuous presence of TTX causes a reversible reduction in spines and increase in filopodia in developing co-cultured SPNs (Segal et al., 2003), and additionally demonstrate that 24 h silencing is sufficient to drive an increase in filopodia along with a strong trend to decreased spine density. Based on previous reports in cortico-striatal co-cultures, SPN spine density increases from ~0.07 to 0.3–0.4 spines/μm between DIV7 and 14 (Burguière et al., 2013; Penrod et al., 2015; Thibault et al., 2016) to ~1 spine/μm by DIV21 (Tian et al., 2010; Penrod et al., 2015). Thus, our reported spine density of ~0.36 and ~0.46 spines/μm at DIV21 following 2× TTX and 3× TTX, respectively, suggest a suppression in the maturing spines between DIV7–21; however, it is also possible that newly formed spines are lost or revert to filopodia upon additional TTX applications. In contrast, given the relative stability of spine densities by three weeks in culture (Penrod et al., 2015), our findings following a 24 h TTX application at DIV20 most likely reflect either a conversion of mature spines to filopodia or distinct regulation of each. Further examination of the mechanisms underlying these changes would benefit from examining SPNs at different developmental stages and at multiple time points following glutamatergic silencing.

That said, the silencing experiments here allow direct comparison of SPN silencing with studies in hippocampal and cortical pyramidal neurons. While a 24 h silencing period in DIV19 hippocampal neurons did not affect spine density, an increase in spine length was interpreted as a possible conversion to filopodia (Papa and Segal, 1996), in support of the results here. Chronic glutamate blockade has yielded somewhat contradictory results elsewhere; one study reported that TTX application throughout synaptogenesis reduced spine density ~50% in cultured hippocampal neurons (Kossel et al., 1997), whereas normal synaptogenesis and spine formation was observed in TTX-treated rat hippocampal slice cultures (McKinney et al., 1999; Soares et al., 2013) and in those from transgenic mice entirely lacking presynaptic glutamate release (Sigler et al., 2017). SPNs also respond differently to disrupted glutamatergic transmission *in vivo*; while hippocampal neurons develop mature spines in the absence of glutamatergic transmission (Sando et al., 2017), reducing glutamatergic release at cortico-striatal synapses in postnatal day (P) eight mice led to a ~40% reduction in spine density measured at P14–15 (Kozorovitskiy et al., 2012). Such changes may be distinct to spiny GABAergic neurons, as GABAergic cerebellar Purkinje cells also lack spines when cultured in the presence of TTX (Schilling et al., 1991). Future comparison of chronic silencing and glutamate receptor blockade in both SPNs and cortical pyramidal neurons in the same co-culture may prove enlightening. This would enable cell-specific spine/filopodia responses to be assessed under the same treatment paradigms and at the same developmental stages.

Intriguingly, we found increased presynaptic Synapsin-1 cluster size and intensity (but not density) following 24 h glutamatergic silencing, in contrast to the decrease observed following the chronic blockade. One possibility for this difference is a transient response to 24 h silencing, related to the immediate pause in the activity-dependent vesicle cycle. Studies of homeostatic plasticity in hippocampal and cortical neurons indicate that ~4–24 h suppression of action potential firing (or AMPAR activity) increases postsynaptic responses (O’Brien et al., 1998; Turrigiano et al., 1998; Stellwagen and Malenka, 2006; Goel and Lee, 2008; Ibata et al., 2008); however, it remains unclear whether this happens in other neuron types (Rutherford et al., 1997; Kim and Tsien, 2009), as does the extent to which homeostatic presynaptic changes in glutamate release and protein expression occur (Erickson et al., 2006; Wierenga et al., 2006; Turrigiano, 2011; Zhao et al., 2011). Although our results indicate that some presynaptic change occurred at cortico-striatal synapses following silencing, which may precede structural changes (as a trend to increased Synapsin-1 cluster size was visible 3 h post-treatment), the interpretation is limited by our choice of presynaptic marker. Given that Synapsin-1 is present at both glutamatergic and GABAergic synapses, we cannot distinguish whether our silencing paradigms differentially altered excitatory or inhibitory input onto SPNs; moreover, opposite regulation of these inputs could cancel one another, or mask additional effects in our readouts. To verify whether the presynaptic input changes observed here were occurring at excitatory synapses, we conducted additional analysis in which we quantified Synapsin-1 puncta on dendritic spines and filopodia only. While this strongly suggests that the observed Synapsin-1 changes were indicative of plasticity at excitatory synapses, future experiments would benefit from staining for synapse-specific pre- and post-synaptic markers to distinguish between inhibitory and excitatory synapses (for example VGLUT1 and PSD95 vs. VGAT and gephyrin, respectively; Rao and Craig, 1997; Levinson and El-Husseini, 2005). This, in combination with electrophysiological recordings of both excitatory and inhibitory post-synaptic currents, would provide more insight as to how different forms of glutamatergic blockade (chronic vs. acute; burst firing vs. receptor blockade) affect both structural and functional plasticity at synapses on SPNs. Future experiments could additionally examine activity-dependant effects on AMPA and NMDA receptor subunit composition and subcellular distribution; given that alterations to these are hallmarks of activity blockade in other glutamatergic neurons (Rao and Craig, 1997; Ehlers, 2003; Soares et al., 2013), it is worth investigating whether similar changes occur in SPNs. Nonetheless, examining the overall change in presynaptic input onto SPNs, in parallel to quantifying dendritic protrusions, highlighted an interesting difference between the response to chronic and 24 h glutamatergic blockade.

Our observations following the additional blockade of AMPARs and NMDARs during 24 h silencing suggest a potential disconnect between the spine and filopodia dynamics. While blocking ionotropic glutamate receptors prevented any suggestion of a change in spine density, a very strong trend to increased filopodia density remained. This is in agreement with results in cortical pyramidal and hippocampal neurons, where NMDAR activity is required for activity-dependent spinogenesis (Fischer et al., 2000; Kwon and Sabatini, 2011) and spine shrinkage/loss (Nägerl et al., 2004; Zhou et al., 2004; Oh et al., 2013), and where AMPAR activity regulates spine motility (Fischer et al., 2000) and maintenance (McKinney et al., 1999). Distinct effects on spine and filopodia dynamics have also been observed elsewhere; blocking AMPA receptors reduced spine density in hippocampal slices 7 days post-treatment, whereas NMDAR blockade had no effect on spines, but instead caused the appearance of filopodia-like protrusions (McKinney et al., 1999).

Beyond methodological variability, different responses to glutamate receptor blockade may arise from the existence of different filopodia sub-types (Portera-Cailliau et al., 2003; Richards et al., 2005), as well as the effects of developmental stage on filopodia dynamics (Sala and Segal, 2014); these distinctions could be tested in future experiments. It is also worth considering that some differences between blocking burst firing vs. all glutamatergic activity may arise from extrasynaptic receptors; while we assume antagonists are most effective in blocking spontaneous glutamate release and activation of receptors close to release sites, we cannot rule out the possibility that silencing extrasynaptic receptor activation through ambient glutamate in the media may contribute. Regardless, our results extend the literature on the effects of glutamatergic receptor blockade to striatal SPNs, demonstrating that, as at other glutamatergic synapses, spine pruning is an active process requiring ongoing low-level glutamate activity, and can be uncoupled from filopodia formation.

The role of NMDARs in striatal activity-dependent plasticity has been revealed primarily by slice electrophysiology (reviewed in Perrin and Venance, 2019). Although LTD was initially considered the dominant form of plasticity at cortico-striatal synapses, many reports have since shown that high-frequency stimulation can result in either NMDAR-dependent LTP, or mGluR-dependent LTD (Calabresi et al., 1996; Spencer and Murphy, 2000; Tang et al., 2001; Reynolds and Wickens, 2002; Wang et al., 2006; Sergeeva et al., 2007; Li et al., 2009; Lovinger and Mathur, 2012; Johnson et al., 2017). However, debate remains as to whether dopamine or other neuromodulators are necessary for the expression of LTP (Spencer and Murphy, 2000; Calabresi et al., 2007; Li et al., 2009; Lovinger, 2010; Burguière et al., 2013; Park et al., 2014; Cerovic et al., 2015). Only a few studies have specifically examined activity-dependent spine alterations in the context of AMPAR trafficking in SPNs (Kozorovitskiy et al., 2012, 2015; Matikainen-Ankney et al., 2018), and, to our knowledge, only one other study has done so in the absence of dopamine (Burguière et al., 2013). Here, we show that LTP-like changes in SPNs can be driven by NMDAR activity alone, as the NMDAR co-agonist glycine (in the absence of Mg^2+^) produced a rapid increase in dendritic spines and associated GluA1 expression. Thus, even in the absence of dopamine, SPNs show a similar response to NMDAR stimulation compared to principle excitatory neurons, in which LTP induction by glutamate uncaging (Matsuzaki et al., 2004; Yang et al., 2008) or chemical paradigms (Lin et al., 2004; Huang et al., 2005; Park et al., 2006; Sharma et al., 2006; Korkotian and Segal, 2007; Fortin et al., 2012; Brigidi et al., 2014) leads to increased spine head volume or *de novo* spine formation without filopodial intermediates (Kwon and Sabatini, 2011). However, a contributing role of non-neurotransmitter neuromodulators within the culture media, such as BDNF, cannot be discounted in the present findings.

The observed increase to GluA1 cluster intensity and association within spines also suggests a functional change, consistent with results in hippocampal neurons showing trafficking and membrane insertion of GluA1-containing AMPARs in spines following LTP induction (Shi et al., 2001; Malinow and Malenka, 2002; Matsuo et al., 2008; Fortin et al., 2010). However, electrophysiological measures of activity-dependent changes here were less clear. Whole-cell patch-clamp recordings ~30–60 min following treatment revealed variable effects on mEPSC frequency, although a trend to increased frequency in glycine-treated SPNs was observed. Moreover, we found no indication of increased mEPSC amplitude following glycine, despite the increased GluA1 signal. A potential reason for this is that the synaptic effects of glycine stimulation are not fully captured by measuring quantal (miniature) glutamatergic transmission, and that changes in evoked activity would be more apparent, given the growing body of literature suggesting that these are mechanistically distinct (Ramirez and Kavalali, 2011; Kavalali, 2015; Abrahamsson et al., 2017; Andreae and Burrone, 2018; Chanaday and Kavalali, 2018). Alternatively, the observed increase in spine density could precede functional changes requiring associated new presynaptic elements (as Synapsin-1 density did not increase); apropos, spine enlargement before AMPAR insertion has been observed following chemical LTP induction in hippocampal slices (Kopec et al., 2006).

In a separate study in which we used the same chemical LTP protocol on cultured hippocampal neurons, we found increased mEPSC amplitude and frequency, which correlated with increased spine width and density 30–60 min after glycine stimulation (Brigidi et al., 2014). It is thus possible that SPNs, unlike hippocampal neurons, require neuromodulators to fully express synaptic LTP in terms of current flux, while structural plasticity can be induced by NMDAR activation alone. In support of this, glutamate uncaging alone leads to spinogenesis in SPNs ~50% of the time, whereas D1 or A2a receptor agonists significantly increase the probability of novel spines and functional synapses (as evidenced by increased mEPSC frequency) in D1R and D2R SPNs, respectively (Kozorovitskiy et al., 2015). Nevertheless, the significant decrease in mEPSC event-decay constant following glycine treatment indicates that some functional change occurred at cortico-striatal synapses, possibly reflecting altered glutamate receptor subunit composition or phosphorylation (Lambolez et al., 1996; Banke et al., 2000; Chater and Goda, 2014). GluA2-lacking, calcium-permeable AMPARs exhibit faster decay kinetics than those containing GluA2 (reviewed in Diering and Huganir, 2018), and multiple studies have reported their integration at specific synapses, including cortico-striatal ones, during LTP induction (Lamsa et al., 2007; Soares et al., 2013; Ma et al., 2018; Park et al., 2018; Benke and Traynelis, 2019). Thus, the faster decay of mEPSCs in glycine-treated co-cultures, together with the increased GluA1 signal in spines, may reflect the activity-dependent insertion of calcium-permeable AMPARs in SPNs. Future work could extend these findings by recording evoked vs. miniature EPSCs, verifying differences in SPN subtype, and/or additionally examining the response to D1 and A2a receptor agonists. Regardless, our results show that NMDAR activation drives rapid structural, and some electrophysiological changes at cortico-striatal synapses.

This study presents an examination of activity-dependent structural development and plasticity within GABAergic striatal projection neurons. Chronic and short-term glutamatergic manipulations to co-cultured SPNs provides a comparison with similar studies in hippocampal and cortical pyramidal neurons and highlights the distinct but overlapping regulation of spine and filopodial activity-dependent plasticity. In particular, we show that SPN structural plasticity occurs within 24 h of glutamate activity blockade, and within 30 min of a 3-min NMDAR activation by glycine, even in the absence of dopamine; thus, the cortico-striatal co-culture system is useful for examining the specific role of glutamate receptor activity in shaping SPN physiology and cortico-striatal synapses. While our primary aim was to provide a characterization of structural plasticity in SPNs and how these may differ from principal excitatory neurons, we offer several ideas on how these assays can be refined and built upon. These could easily be applied to examining activity-dependent plasticity in disease models, particularly those in which altered glutamatergic transmission and aberrant structural plasticity may play a pathophysiological role, and in which SPNs have shown distinct vulnerability.

## Data Availability Statement

The raw data supporting the conclusions of this article will be made available by the authors upon request, without undue reservation.

## Ethics Statement

The animal study was reviewed and approved by Canadian Council on Animal Care.

## Author Contributions

NK and AM designed the study and co-wrote the manuscript. With supervision from AM, NK conducted all experiments, analyzed data, interpreted results, and made figures. MW and LQ-R helped with image processing and analysis. MF provided scientific input and supervisory support throughout. All authors contributed to the article and approved the submitted version.

## Conflict of Interest

The authors declare that the research was conducted in the absence of any commercial or financial relationships that could be construed as a potential conflict of interest.

## Supplementary Material

SUPPLEMENTARY FIGURE 1 Synapsin-1 total dendritic density, and spine-/filopodia-specific changes in Synapsin-1 puncta following chronic and acute silencing. Additional analysis to quantify the effects of glutamatergic silencing on Synapsin-1 puncta at select dendritic protrusions as opposed to the full dendritic arbor. In a subset of images (5–10 per culture) in chronic **(A)** and acute **(B)** silencing experiments, ROIs were created around a sample of spines and any visible filopodia on secondary or tertiary dendrites of GFP-expressing SPNs, to calculate the integrated density (intensity) of Synapsin-1 in the corresponding raw images. **(A)** There were no changes in total dendritic Synapsin-1 cluster density in chronic TTX silencing experiments; analysis of dendritic spines and filopodia showed a significant reduction in Synapsin-1 integrated intensity on spines following silencing (Kruskal–Wallis test, *****p* < 0.0001), with post-hoc tests indicating that the 3× TTX condition was reduced compared to the 2× TTX condition and control (Uncorrected Dunn’s test; ***p* = 0.008 and *****p* < 0.0001, respectively), whereas there was no significant difference between 2× TTX and control (*p* = 0.291). In contrast, Synapsin-1 signal on filopodia was significantly higher in the 2× TTX condition when compared to control (Kruskal–Wallis test, ****p* = 0.0008; *post-hoc* Uncorrected Dunn’s test, **p* = 0.034) and compared to the 3× TTX condition (****p* = 0.0002). **(B)** Acute (24 h) silencing experiments, with separate analysis for dendritic spines and filopodia. There were no changes in total dendritic Synapsin-1 cluster density in 24 h acute TTX silencing experiments. On spines, both the Silencing (TTX only) and Total Silencing (TTX + APV + CNQX) conditions showed significantly increased Synapsin-1 integrated intensity compared to control (Kruskal–Wallis test, *****p* < 0.0001; *post-hoc* Uncorrected Dunn’s Test, *****p* < 0.0001 for both). Additionally, the Total Silencing condition had a significantly greater increase compared to the Silencing condition (****p =* 0.0003). In contrast, there was no significant difference in Synapsin-1 signal on filopodia (Kruskal–Wallis test, *p* = 0.838).

## Abbreviations

SPN: striatal projection neuron
D1R: Drd1 dopamine receptor
D2R: Drd2 dopamine receptor
LTP: long-term potentiation
LTD: long-term depression
TTX: tetrodotoxin
DIV: days *in vitro*
AP5: D-(−)-2-Amino-5-phosphonopentanoic acid
CNQX: 6-cyano-7-nitroquinoxaline-2, 3-dione disodium salt
mEPSC: miniature excitatory post-synaptic current
cLTP: chemical LTP
PTX: picrotoxin
NGS: normal goat serum
PBS: phosphate-buffered saline
RT: room temperature.
