# Supplementary figures and images for: Chronic and Acute Manipulation of Cortical Glutamate Transmission Induces Structural and Synaptic Changes in Co-cultured Striatal Neurons
Source: Front Cell Neurosci. 2021 Feb 18;15:569031. doi: 10.3389/fncel.2021.569031 (PMC7930618; doi:10.3389/fncel.2021.569031)

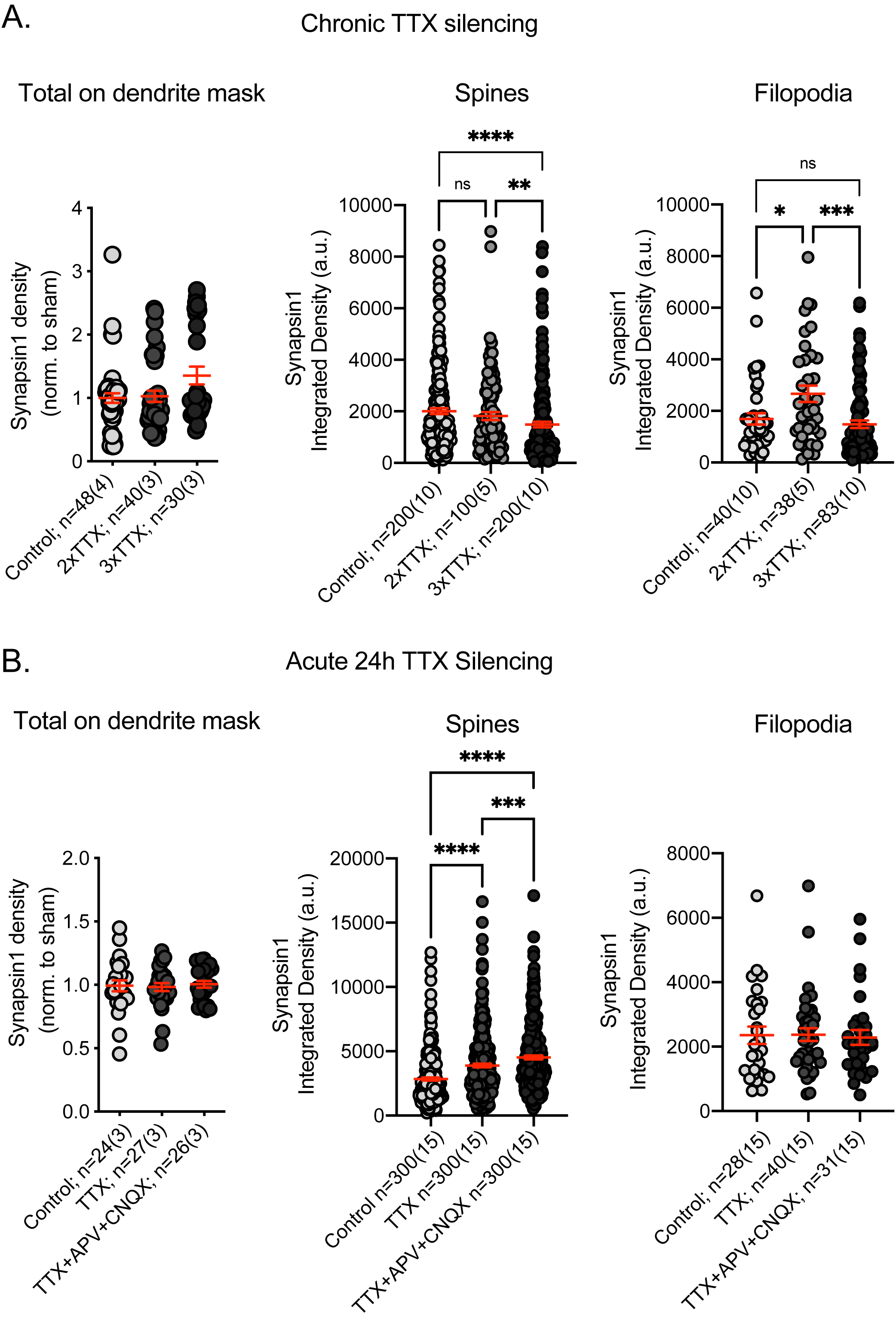

Supplement: Supplementary file 1 [file Image_1.JPEG]
