# Supplementary material for: Chronic and Acute Manipulation of Cortical Glutamate Transmission Induces Structural and Synaptic Changes in Co-cultured Striatal Neurons
Source: Front Cell Neurosci. 2021 Feb 18;15:569031. doi: 10.3389/fncel.2021.569031 (PMC7930618; doi:10.3389/fncel.2021.569031)
Supplement: SUPPLEMENTARY FIGURE 1 — Synapsin-1 total dendritic density, and spine-/filopodia-specific changes in Synapsin-1 puncta following chronic and acute silencing. Additional analysis to quantify the effects of glutamatergic silencing on Synapsin-1 puncta at select dendritic protrusions as opposed to the full dendritic arbor. In a subset of images (5–10 per culture) in chronic (A) and acute (B) silencing experiments, ROIs were created around a sample of spines and any visible filopodia on secondary or tertiary dendrites of GFP-expressing SPNs, to calculate the integrated density (intensity) of Synapsin-1 in the corresponding raw images. (A) There were no changes in total dendritic Synapsin-1 cluster density in chronic TTX silencing experiments; analysis of dendritic spines and filopodia showed a significant reduction in Synapsin-1 integrated intensity on spines following silencing (Kruskal–Wallis test, ****p < 0.0001), with post-hoc tests indicating that the 3× TTX condition was reduced compared to the 2× TTX condition and control (Uncorrected Dunn’s test; **p = 0.008 and ****p < 0.0001, respectively), whereas there was no significant difference between 2× TTX and control (p = 0.291). In contrast, Synapsin-1 signal on filopodia was significantly higher in the 2× TTX condition when compared to control (Kruskal–Wallis test, ***p = 0.0008; post-hoc Uncorrected Dunn’s test, *p = 0.034) and compared to the 3× TTX condition (***p = 0.0002). (B) Acute (24 h) silencing experiments, with separate analysis for dendritic spines and filopodia. There were no changes in total dendritic Synapsin-1 cluster density in 24 h acute TTX silencing experiments. On spines, both the Silencing (TTX only) and Total Silencing (TTX + APV + CNQX) conditions showed significantly increased Synapsin-1 integrated intensity compared to control (Kruskal–Wallis test, ****p < 0.0001; post-hoc Uncorrected Dunn’s Test, ****p < 0.0001 for both). Additionally, the Total Silencing condition had a significantly greater increase compared t [file Data_Sheet_1.pdf]

```
CellProfiler Pipeline: http://www.cellprofiler.org
Version:1
SVNRevision:11710

LoadImages:[module_num:1|svn_version:\'11587\'|
variable_revision_number:11|show_window:False|notes:\x5B"Load each
channel (or stain) as a separate image.  If you have a color image
composed of different stains, you\'ll need a ColorToGray module after
LoadImages to separate the incoming image into its component
channels.", \'\', \'In the example pipeline, we call the two images
OrigStain1 and OrigStain2.\'\x5D]
    File type to be loaded:individual images
    File selection method:Text-Exact match
    Number of images in each group?:3
    Type the text that the excluded images have in common:Do not use
    Analyze all subfolders within the selected folder?:None
    Input image file location:Default Input Folder\x7C
    Check image sets for missing or duplicate files?:Yes
    Group images by metadata?:No
    Exclude certain files?:No
    Specify metadata fields to group by:
    Select subfolders to analyze:
    Image count:3
    Text that these images have in common (case-sensitive):Bsynapsin-
    Position of this image in each group:1
    Extract metadata from where?:None
    Regular expression that finds metadata in the file name:^(?
P<Plate>.+)_(?P<WellRow>\x5BA-P\x5D)(?
P<WellColumn>\x5B0-9\x5D{1,2})_(?P<Site>\x5B0-9\x5D)
    Type the regular expression that finds metadata in the subfolder
path:(?P<Year>\x5B0-9\x5D{4})-(?P<Month>\x5B0-9\x5D{2})-(?
P<Day>\x5B0-9\x5D{2})
    Channel count:1
    Group the movie frames?:No
    Grouping method:Interleaved
    Number of channels per group:2
    Load the input as images or objects?:Images
    Name this loaded image:syn Mask
    Name this loaded object:Nuclei
    Retain outlines of loaded objects?:No
    Name the outline image:NucleiOutlines
    Channel number:1
    Rescale intensities?:Yes
    Text that these images have in common (case-sensitive):MGFP-
    Position of this image in each group:3
    Extract metadata from where?:None
    Regular expression that finds metadata in the file name:^(?
P<Plate>.*)_(?P<Well>\x5BA-P\x5D\x5B0-9\x5D{2})_s(?P<Site>\x5B0-9\x5D)
    Type the regular expression that finds metadata in the subfolder
path:.*\x5B\\\\/\x5D(?P<Date>.*)\x5B\\\\/\x5D(?P<Run>.*)$
```

```
    Channel count:1
    Group the movie frames?:No
    Grouping method:Interleaved
    Number of channels per group:3
    Load the input as images or objects?:Images
    Name this loaded image:GFPSTAIN-
    Name this loaded object:Nuclei
    Retain outlines of loaded objects?:No
    Name the outline image:LoadedImageOutlines
    Channel number:1
    Rescale intensities?:Yes
    Text that these images have in common (case-sensitive):Usynapsin-
    Position of this image in each group:4
    Extract metadata from where?:None
    Regular expression that finds metadata in the file name:^(?
P<Plate>.*)_(?P<Well>\x5BA-P\x5D\x5B0-9\x5D{2})_s(?P<Site>\x5B0-9\x5D)
    Type the regular expression that finds metadata in the subfolder
path:.*\x5B\\\\/\x5D(?P<Date>.*)\x5B\\\\/\x5D(?P<Run>.*)$
    Channel count:1
    Group the movie frames?:No
    Grouping method:Interleaved
    Number of channels per group:3
    Load the input as images or objects?:Images
    Name this loaded image:syn
    Name this loaded object:Nuclei
    Retain outlines of loaded objects?:No
    Name the outline image:LoadedImageOutlines
    Channel number:1
    Rescale intensities?:Yes

IdentifyPrimaryObjects:[module_num:2|svn_version:\'10826\'|
variable_revision_number:8|show_window:True|notes:\x5B\x5D]
    Select the input image:GFPSTAIN-
    Name the primary objects to be identified:Cell
    Typical diameter of objects, in pixel units (Min,Max):50,1000
    Discard objects outside the diameter range?:Yes
    Try to merge too small objects with nearby larger objects?:Yes
    Discard objects touching the border of the image?:No
    Select the thresholding method:Otsu Global
    Threshold correction factor:1
    Lower and upper bounds on threshold:0.000000,1.000000
    Approximate fraction of image covered by objects?:0.01
    Method to distinguish clumped objects:None
    Method to draw dividing lines between clumped objects:Intensity
    Size of smoothing filter:10
    Suppress local maxima that are closer than this minimum allowed
distance:7
    Speed up by using lower-resolution image to find local maxima?:Yes
    Name the outline image:PrimaryOutlines
    Fill holes in identified objects?:No
```

```
    Automatically calculate size of smoothing filter?:Yes
    Automatically calculate minimum allowed distance between local maxima?:Yes
    Manual threshold:0.0
    Select binary image:None
    Retain outlines of the identified objects?:No
    Automatically calculate the threshold using the Otsu method?:Yes
    Enter Laplacian of Gaussian threshold:0.5
    Two-class or three-class thresholding?:Two classes
    Minimize the weighted variance or the entropy?:Weighted variance
    Assign pixels in the middle intensity class to the foreground or the background?:Foreground
    Automatically calculate the size of objects for the Laplacian of Gaussian filter?:Yes
    Enter LoG filter diameter:5
    Handling of objects if excessive number of objects identified:Continue
    Maximum number of objects:500
    Select the measurement to threshold with:None

MeasureObjectSizeShape:[module_num:3|svn_version:\'1\'|variable_revision_number:1|show_window:False|notes:\x5B\x5D]
    Select objects to measure:Cell
    Calculate the Zernike features?:No

ExportToSpreadsheet:[module_num:4|svn_version:\'10880\'|variable_revision_number:7|show_window:False|notes:\x5B\x5D]
    Select or enter the column delimiter:Comma (",")
    Prepend the output file name to the data file names?:Yes
    Add image metadata columns to your object data file?:No
    Limit output to a size that is allowed in Excel?:No
    Select the columns of measurements to export?:No
    Calculate the per-image mean values for object measurements?:No
    Calculate the per-image median values for object measurements?:No
    Calculate the per-image standard deviation values for object measurements?:No
    Output file location:Default Output Folder\x7CNone
    Create a GenePattern GCT file?:No
    Select source of sample row name:Metadata
    Select the image to use as the identifier:None
    Select the metadata to use as the identifier:None
    Export all measurements, using default file names?:Yes
    Press button to select measurements to export:
    Data to export:Do not use
    Combine these object measurements with those of the previous object?:No
    File name:DATA.csv
    Use the object name for the file name?:Yes

ExpandOrShrinkObjects:[module_num:5|svn_version:\'10830\'|
```

```
variable_revision_number:1|show_window:False|notes:\x5B\x5D]
    Select the input objects:Cell
    Name the output objects:Expanded GFP
    Select the operation:Expand objects by a specified number of
pixels
    Number of pixels by which to expand or shrink:5
    Fill holes in objects so that all objects shrink to a single
point?:No
    Retain the outlines of the identified objects for use later in the
pipeline (for example, in SaveImages)?:No
    Name the outline image:ShrunkenNucleiOutlines

IdentifyPrimaryObjects:[module_num:6|svn_version:\'10826\'|
variable_revision_number:8|show_window:True|notes:\x5B\'Similarly,
correlation measurements for individual objects can also be obtained.
However, to determine colocalization on per-object basis, the objects
within the image must be identified.  We first segment the image
features into objects, then make comparisons between the individual
objects in the channels.\', \'\', \'The input image is selected as
Stain1, with the output objects named Objects1. The typical diameter
is set as \x5B3,15\x5D for the min/max size we expect the objects to
be. We chose to discard small and large objects, which tend to be
spurious, and discard those objects at the border because we will be
making area-based measurements.\', \'\', \'The chosen thresholding
method can greatly affect segmentation.  Here, you want to select a
method that will accurately identify the protein of interest as
foreground.  Depending on the background level and properties of the
stain, you may need to try several different methods and corresponding
settings to obtain good segmentation.  Please see the help for
IdentifyPrimaryObjects for more information on the thresholding
methods available.\', \'\', \'Settings to distinguish clumped objects
are of importance for per-object measures of co-localization.  For
example, if you wish to measure co-localization only in the nuclei or
cytoplasm, each sub cellular compartment must be properly segmented to
provide an accurate measurement.  You may need to adjust various
settings to get good segmentation of clumpy nuclei. \'\x5D]
    Select the input image:syn Mask
    Name the primary objects to be identified:syn puncta
    Typical diameter of objects, in pixel units (Min,Max):4,15
    Discard objects outside the diameter range?:Yes
    Try to merge too small objects with nearby larger objects?:No
    Discard objects touching the border of the image?:No
    Select the thresholding method:Otsu Global
    Threshold correction factor:1.0
    Lower and upper bounds on threshold:0.0005,1.0
    Approximate fraction of image covered by objects?:0.01
    Method to distinguish clumped objects:Intensity
    Method to draw dividing lines between clumped objects:Intensity
    Size of smoothing filter:10
    Suppress local maxima that are closer than this minimum allowed
```

```
distance:7
    Speed up by using lower-resolution image to find local maxima?:Yes
    Name the outline image:Stain1Outlines
    Fill holes in identified objects?:Yes
    Automatically calculate size of smoothing filter?:Yes
    Automatically calculate minimum allowed distance between local
maxima?:Yes
    Manual threshold:0.0
    Select binary image:None
    Retain outlines of the identified objects?:Yes
    Automatically calculate the threshold using the Otsu method?:Yes
    Enter Laplacian of Gaussian threshold:0.5
    Two-class or three-class thresholding?:Two classes
    Minimize the weighted variance or the entropy?:Weighted variance
    Assign pixels in the middle intensity class to the foreground or
the background?:Background
    Automatically calculate the size of objects for the Laplacian of
Gaussian filter?:Yes
    Enter LoG filter diameter:5
    Handling of objects if excessive number of objects
identified:Continue
    Maximum number of objects:500
    Select the measurement to threshold with:None

MaskImage:[module_num:7|svn_version:\'10428\'|
variable_revision_number:3|show_window:True|notes:\x5B\x5D]
    Select the input image:syn
    Name the output image:masked syn
    Use objects or an image as a mask?:Objects
    Select object for mask:syn puncta
    Select image for mask:None
    Invert the mask?:No

MeasureObjectIntensity:[module_num:8|svn_version:\'10816\'|
variable_revision_number:3|show_window:False|notes:\x5B\x5D]
    Hidden:1
    Select an image to measure:masked syn
    Select objects to measure:syn puncta

MeasureObjectSizeShape:[module_num:9|svn_version:\'1\'|
variable_revision_number:1|show_window:True|notes:\x5B\x5D]
    Select objects to measure:syn puncta
    Calculate the Zernike features?:Yes

ExportToSpreadsheet:[module_num:10|svn_version:\'10880\'|
variable_revision_number:7|show_window:False|notes:\x5B\'This module
is used to export the full set of measurements obtained by the
pipeline. Measurements such as object counts, colocalization
percentages and area fractions are saved to a per-image file (that is,
one value per image); measurements such as colocalized/non-colocalized
```

```
status and centroid distances are saved to a per-object file (one
value per object).\'\x5D]
    Select or enter the column delimiter:Comma (",")
    Prepend the output file name to the data file names?:Yes
    Add image metadata columns to your object data file?:No
    Limit output to a size that is allowed in Excel?:No
    Select the columns of measurements to export?:No
    Calculate the per-image mean values for object measurements?:No
    Calculate the per-image median values for object measurements?:No
    Calculate the per-image standard deviation values for object
measurements?:No
    Output file location:Default Output Folder\x7C.
    Create a GenePattern GCT file?:No
    Select source of sample row name:Metadata
    Select the image to use as the identifier:None
    Select the metadata to use as the identifier:None
    Export all measurements, using default file names?:No
    Press button to select measurements to export:None\x7CNone
    Data to export:Image
    Combine these object measurements with those of the previous
object?:No
    File name:Image_DATA.csv
    Use the object name for the file name?:Yes

MaskImage:[module_num:11|svn_version:\'10428\'|
variable_revision_number:3|show_window:True|notes:\x5B\x5D]
    Select the input image:syn Mask
    Name the output image:GFP masked syn mask
    Use objects or an image as a mask?:Objects
    Select object for mask:Expanded GFP
    Select image for mask:None
    Invert the mask?:No

IdentifyPrimaryObjects:[module_num:12|svn_version:\'10826\'|
variable_revision_number:8|show_window:True|notes:\x5B\x5D]
    Select the input image:GFP masked syn mask
    Name the primary objects to be identified:syn puncta in GFP
    Typical diameter of objects, in pixel units (Min,Max):4,15
    Discard objects outside the diameter range?:Yes
    Try to merge too small objects with nearby larger objects?:No
    Discard objects touching the border of the image?:No
    Select the thresholding method:Otsu Global
    Threshold correction factor:1.0
    Lower and upper bounds on threshold:0.0005,1.0
    Approximate fraction of image covered by objects?:0.01
    Method to distinguish clumped objects:Intensity
    Method to draw dividing lines between clumped objects:Intensity
    Size of smoothing filter:10
    Suppress local maxima that are closer than this minimum allowed
distance:7
```

```
    Speed up by using lower-resolution image to find local maxima?:Yes
    Name the outline image:Stain1Outlines
    Fill holes in identified objects?:Yes
    Automatically calculate size of smoothing filter?:Yes
    Automatically calculate minimum allowed distance between local
maxima?:Yes
    Manual threshold:0.0
    Select binary image:None
    Retain outlines of the identified objects?:Yes
    Automatically calculate the threshold using the Otsu method?:Yes
    Enter Laplacian of Gaussian threshold:0.5
    Two-class or three-class thresholding?:Two classes
    Minimize the weighted variance or the entropy?:Weighted variance
    Assign pixels in the middle intensity class to the foreground or
the background?:Background
    Automatically calculate the size of objects for the Laplacian of
Gaussian filter?:Yes
    Enter LoG filter diameter:5
    Handling of objects if excessive number of objects
identified:Continue
    Maximum number of objects:500
    Select the measurement to threshold with:None

MaskImage:[module_num:13|svn_version:\'10428\'|
variable_revision_number:3|show_window:True|notes:\x5B\x5D]
    Select the input image:syn
    Name the output image:masked syn in GFP
    Use objects or an image as a mask?:Objects
    Select object for mask:syn puncta in GFP
    Select image for mask:None
    Invert the mask?:No

MeasureObjectIntensity:[module_num:14|svn_version:\'10816\'|
variable_revision_number:3|show_window:False|notes:\x5B\x5D]
    Hidden:1
    Select an image to measure:masked syn in GFP
    Select objects to measure:syn puncta in GFP

MeasureObjectSizeShape:[module_num:15|svn_version:\'1\'|
variable_revision_number:1|show_window:True|notes:\x5B\x5D]
    Select objects to measure:syn puncta in GFP
    Calculate the Zernike features?:Yes

ExportToSpreadsheet:[module_num:16|svn_version:\'10880\'|
variable_revision_number:7|show_window:False|notes:\x5B\x5D]
    Select or enter the column delimiter:Comma (",")
    Prepend the output file name to the data file names?:Yes
    Add image metadata columns to your object data file?:No
    Limit output to a size that is allowed in Excel?:No
    Select the columns of measurements to export?:No
```

```
    Calculate the per-image mean values for object measurements?:No
    Calculate the per-image median values for object measurements?:No
    Calculate the per-image standard deviation values for object
measurements?:No
    Output file location:Default Output Folder\x7CNone
    Create a GenePattern GCT file?:No
    Select source of sample row name:Metadata
    Select the image to use as the identifier:None
    Select the metadata to use as the identifier:None
    Export all measurements, using default file names?:Yes
    Press button to select measurements to export:
    Data to export:Do not use
    Combine these object measurements with those of the previous
object?:No
    File name:DATA.csv
    Use the object name for the file name?:Yes
```
